# Utility of Plasmapheresis in Autoimmune-Mediated Encephalopathy in Children: Potentials and Challenges

**DOI:** 10.1155/2016/7685807

**Published:** 2016-04-28

**Authors:** Abdulhafeez M. Khair

**Affiliations:** Department of Pediatrics, Division of Pediatric Neurology, Hamad Medical Corporation, Doha, Qatar

## Abstract

Autoimmune-mediated encephalopathy in children continues to constitute a diagnostic and therapeutic challenge in pediatric population. Utility and usefulness in this clinical setting of plasmapheresis have seldom been evaluated in current pediatric literature. Children with immune-mediated encephalopathies represent a uniquely different group among patients presenting to intensive care units or neurological services worldwide. Arriving at a final diagnosis is not an easy task for treating physicians. It is very crucial to consider early use of first-line immunotherapy modalities, save those children's lives and improve outcomes. Plasmapheresis is an emerging, potentially beneficial first-line therapy in such patients. However, indications, value, logistics, and procedural difficulties are often faced. This study is mainly meant to review the current knowledge in regard to the clinical value of plasmapheresis in children with immune-mediated encephalopathy.

## 1. Introduction

Encephalopathy is a commonly used terminology despite having very vague definition and quite nonspecific application. The term in fact can be applied to any diffuse disease of the brain that alters brain function or structure [1]. The term encephalitis is usually used when encephalopathy (more precisely altered mental status) is accompanied by more than one of the following clinical manifestations: fever, seizures, focal neurological signs, cerebrospinal fluid (CSF) pleocytosis, or abnormal brain neuroimaging studies [2]. The etiological list of causes of encephalopathy or encephalitis is quite long, yet the main focus of this study is autoimmune encephalopathy (AIE) in the pediatric age group.

## 2. AIE Clinical Complexity

The spectrum of AIE cannot be dealt with in isolation in regard to other autoimmune phenomena, forming a foundation for many intersecting childhood illnesses. Diagnosis of AIE is not without challenges. Close similarity with many forms of infectious and inflammatory encephalitis can be a major obstacle in identifying children with AIE [3]. Wide variability of possible panels of autoimmune antibodies, logistics in regard to technicalities and cost of those sophisticated tests, and poor clinical specificity are among additional difficulties as well.

The clinical phenotype is extensively diverse. The same diversity scale applies to the underlying pathophysiological mechanisms as well. In fact, the clinical spectrum of pediatric AIE can be expanded to include various clinical entities and neurological syndromes like poststreptococcal encephalopathy, limbic encephalitis, acute demyelinating encephalomyelitis (ADEM), anti-voltage-gated potassium channels (VGKC-mediated) encephalitis, alpha amino 3-hydroxyl 5-methyl 4-isoxazolepropionic acid receptor (AMPAR) encephalitis, gamma amino butyric acid (GABA) encephalitis, the Rasmussen encephalitis, glycine receptor encephalitis, and many others [4].

Harmonizing a standard pathway for diagnosis of AIE is difficult. Some clues that might help to entertain the possibility of AIE as a diagnosis are atypical clinical presentation, low inflammatory markers, failure to isolate a causative organism, poor response to antimicrobial agents, and suspicion of underlying malignancy. Ominous neuropsychiatric symptoms might draw attention to AIE in the setting of encephalopathy if no other explanation can be reached. Severely disturbed sleep cycle is a frequent observation in children with AIE [5]. Several forms of movement disorders, specially oral and facial dyskinetic movements, are seen more often in pediatric AIE as compared to infectious encephalitis [5]. In the absence of specificity of any of the above clinical data, considering both workup and active management simultaneously might be warranted to improve the clinical outcome.

## 3. AIE Epidemiology and Etiology

Although AIE is considered a rather rare disease, it is believed that children represent up to 40% of the total cohort of patients. Seventy-five children have been followed in a big multicenter prospective study in United Kingdom [6]. In one-third of this UK cohort, the diagnosis of AIE has never been surely confirmed [6]. Moreover, nearly half of the patients tested negative for the known common autoimmune antibodies [4]. A more recent and bigger study is the California encephalitis project [7]. In this relatively large epidemiological study almost 1500 patients (adults and children) have been involved. In this cohort, confirmed or strongly suspected etiologies were found in only one-third of the study population, while nearly two-thirds have remained with unknown etiology [7]. In adults, a good proportion of patients with AIE are attributable to a paraneoplastic phenomenon, and anti-Hu antibodies are probably the most standing out in this group [8, 9]. These antibodies are believed to be directed against neuron specific RNA-binding nuclear proteins. Another related group is the cohort of adults with paraneoplastic anti-LGI1 limbic encephalitis, classically described with small lung carcinomas [10]. This kind of overt paraneoplastic correlation seems to be less obvious in pediatric age group though. In a European multicenter study, none of the ten pediatric patients with confirmed AIE has any evidence of associated malignancy [11].

## 4. Anti-N-methyl-D-aspartate (Anti-NMDA) Receptor Encephalopathy

Anti-NMDA receptor antibodies disease remains the main prototype of AIE in children and young adults under 18 years of age [12], excluding the patients with classical ADEM. Although it tends to occur in older children, it has been described in young children, including infants [13]. A constellation mix of seizures, behavioral changes, and various types of nonepileptic movements is the hallmark of the clinical presentation [14]. Memory loss, sleep disturbance, overt psychosis, speech disorder, altered sensorium, and orofacial dyskinesia are among the commonest clinical clues as well [15]. Fortunately, and unlike adults, autonomic disturbances and ventilation difficulties are quite exceptional [16]. Interestingly, CSF alterations, reversible magnetic resonance imaging (MRI) changes, and electrical slowing in electroencephalogram (EEG) seem to be of no significant difference among patients who have tested positive versus negative for the anti-NMDA antibody titers [16].

## 5. Treatment Strategies for Pediatric AIE

Removing the correlating neoplasm (if present) and utilizing immunotherapy modalities are the most efficient strategies in treating children with AIE [8]. Nevertheless, isolating a solid tumor in children with AIE is seldom successful. Supportive care, dietary support, sleep aid, and antiseizure and antimovement therapies are all essential in complementing the healthcare of those patients [17]. First-line immunotherapy tools are high dose steroid therapy, intravenous immunoglobulins, and possibly exchange plasmapheresis. There is growing evidence eluding other immune-modulators such as rituximab and cyclophosphamide being beneficial as well [18, 19]. What is still unclear is the exact response to those treatment modalities on the long run; however a quarter to one-third of patients are expected to have rapid improvement and complete recovery [20]. From few available prospective follow-up studies, children are expected to continue improving even after months to years [21] after proper immunotherapy. This observation is probably stressing on the need for long term follow-up. This is particularly important in regard to patients and families' counseling. Interestingly, some children attain full recovery in spite of highly detectable autoantibody titers [22]. It is not known at the moment whether this is the reason behind some few relapses in those patients [23].

## 6. Plasmapheresis in Pediatric AIE

As far as plasmapheresis is concerned, literature is obviously deficient in regard to indications, efficiency, technical operations, and possible disadvantages. There are neither randomized trials in pediatric patients nor head-to-head studies for comparison with other immunotherapies. However, there is good evidence that plasmapheresis, as the case with other immunotherapies, should be considered as early as the diagnosis of AIE is entertained, in order to improve the final outcome [20]. Plasmapheresis seems to be effective as a first-line immunotherapy [24], although there are no side-to-side controlled studies against immunoglobulins or corticosteroids [25]. Nevertheless, the American Society for Apheresis has considered plasmapheresis to be classified as category 3 grade 2C evidence for treatment of confirmed autoimmune diseases [26]. Having said that, preliminary data are showing that corticosteroids alone may not be as effective as steroids followed by plasmapheresis, though these very novel results are yet to be concluded [27]. The author is not aware of evidence-based recommendation in regard to plasmapheresis number and volume of sessions. Although the consensus-based recommendations are often four to six sessions of large volume plasmapheresis, using small volumes twice daily for seven to ten days might serve an alternative option if complications with large volumes removal are likely to be encountered [28].

## 7. Plasmapheresis Procedural Technicalities

Although detailed technical description is outside the scope of this review, it is always helpful to understand the basic procedure principles. A prerequisite set of laboratory investigations is usually carried out, including full blood counts, electrolyte, and parameters of coagulation profile. Central line is flushed and tubes are primed with packed red blood cells or normal saline. Blood and plasma volumes are estimated according to age, weight, and height of the patient. The replacing plasma volume and speed are then calculated. Blood is drawn through draw lumen to be centrifuged; plasma part is discarded whereas packed cells are recirculated back to the patient along with the fresh new plasma. Heparin is kept in lumen after finishing the session to prevent clot formation. Monitoring is needed after plasmapheresis to ensure safe clinical status and normal laboratory readings, usually in pediatric intensive care settings.

## 8. Plasmapheresis in Anti-NMDA AIE

Smith and his group have reported a young adult with proven anti-NMDA receptor encephalitis with severe presentation and opsoclonus-myoclonus, who failed to respond to high dose corticosteroids but responded dramatically to plasmapheresis [29]. Pham and his group have followed a larger group of nine pediatric patients with anti-NMDA AIE who underwent alternate day regimen of plasmapheresis, with only one patient having immediate improvement, whereas three continued to improve after discharge [30]. Of interest, in his study Pham has noticed better and faster improvement in patients who received immunoglobulins after plasmapheresis in comparison to those who received it before [30]. Ishiura and his colleagues from Japan have reported a successful combination of plasmapheresis and rituximab in an adult patient [31]. Agrawal and his group have also reported a successful treatment of a young two-year-old child with postvaccination anti-NMDA AIE after long period of prior treatment-resistance [32]. This recently reported child had twenty cycles of plasmapheresis after four months of illness in combination with immunosuppressant therapy with Mycophenolate, with ultimate good outcome and satisfying developmental catch-up. The same group has reported that another twelve-year-old girl with catatonia and encephalopathy, who had proven anti-NMDA AIE, arrived into a full clinical recovery after plasmapheresis [33]. Occasionally isolating the autoantibodies is the discovery that alerts physician to the diagnosis of AIE, thus considering plasmapheresis for quicker recovery [34]. Although time factor seems to be crucial for the desired outcome, plasmapheresis has been tried as a second-line therapy with good success in some reports as well [35].

## 9. Plasmapheresis in ADEM/Transverse Myelitis (TM)

Plasmapheresis utilization in managing children with ADEM is better established in current literature [36–38]. Moreover, plasmapheresis might be of benefit even in the more severe cases; those are usually unresponsive to immunoglobulins or steroid therapies [39, 40]. In spite of lack of face-to-face studies once again, plasmapheresis was found to be beneficial when used in combination with high dose corticosteroids compared to single therapy in patients with idiopathic TM [41]. It is still unclear though whether this statistical observation is applicable to patients with classical ADEM. A closely related disorder is neuromyelitis optica spectrum (NMO), a rare immune-mediated neurological disorder presenting mainly with combined features of TM and optic neuritis [42]. In contrast to adults with NMO, pediatric patients appear to have better response to plasmapheresis in the steroid-resistant groups [43]. In spite of such promising observation, up to a quarter of patients might still show no response regardless of the number of plasmapheresis sessions received [44].

## 10. Plasmapheresis in Hashimoto's Encephalopathy (HE)

HE is a rare yet devastating disorder which is a considered a variety of AIE. Most patients are adults who usually present with rapidly progressive dementia, altered sensorium, hallucinations, and various seizure types. Levels of thyroglobulin and thyroperoxidase are typically high. The disease has been described classically as steroid-responsive in pediatric age [45]. Nevertheless, plasmapheresis often helps in normalizing the antibodies' titers and hence significant clinical improvement [46]. The first pediatric patient with HE to be treated with plasmapheresis has been reported recently by Bektas and his group in Turkey [47]. In their study they have reported a 12-year-old boy with refractory epileptic seizures and high anti-thyroid peroxidase antibodies and negative for other autoantibodies, who responded dramatically to plasmapheresis, both clinically and in laboratory [47].

## 11. Plasmapheresis in Neuropsychiatric Lupus (NL)

There are other variants of pediatric AIE in which plasmapheresis has been proposed but there is no enough supportive evidence at the moment. Neuropsychiatric lupus (NL) is a rare immune-mediated encephalopathy affecting around one-third of patients with systemic lupus (SLE). The severity of symptoms varies, but the effect on quality of life is often tremendous. Most patients demonstrate significant behavioral and psychiatric manifestation. In severe cases of NL, plasmapheresis has been used as an adjuvant therapy with cyclophosphamide, resulting in complete remission in five out of 10 patients in one retrospective study [48]. In another review, twenty-six patients with mix of adults and children with NL have received plasmapheresis with 74% clinical response rate [49].

## 12. Plasmapheresis in Poststreptococcal Encephalopathy

Pediatric autoimmune neuropsychiatric disorders associated with streptococcus infection (PANDAS) represent an interesting subgroup of AIE in the sense that many scientists do either believe or disbelieve in its existence as a separate disease entity. The clinical symptoms may include tic movement, obsessive compulsive tantrums, anxiety, sleep disruptions, variable behavioral changes, and even Frank psychosis [50]. Formulation of agreed diagnostic criteria has been a difficult task till some have been published recently [51]. The first trial of plasmapheresis in PANDAS was in 1999 by Perlmutter, who reported 10 pediatric patients with PANDAS who had showed superior response to plasmapheresis compared to placebo in terms of severity of tics, obsessive-compulsive scale scores [52]. Few years later, Besiroglu and his group have reported four adults with adult-variant PANDAS with near complete recovery after receiving repeated sessions of plasmapheresis [53]. The biggest pediatric series however has just been published by Latimer and his group. In this review of 35 patients, plasmapheresis has proved to be safe, efficient, and well tolerated in severe cases of PANDAS [54]. A cousin disorder of poststreptococcal origin is Sydenham's chorea (SC) but is often more benign and self-limiting. Being assumed of autoimmune nature, immunotherapy for SC has been tried years ago [55]. Response to plasmapheresis has been occasionally reported to be dramatic, even in the very severe disabling pediatric cases [56]. It is worth mentioning that American Academy of Neurology (AAN) has described in its last recommendations the evidence behind usage of plasmapheresis in PANDAS and Sydenham's chorea as insufficient [57].

## 13. Plasmapheresis in the Rasmussen Encephalitis (RE)

RE is a severe form of encephalopathy in children, manifesting as progressive cognitive decline with characteristic refractory focal seizures and focal neurological deficits [58]. Several underlying autoantibodies have been suggested as etiological chemicals. Response to treatment to immunotherapy modalities is often limited and short-lived [59]. Rarely a sustainable improvement can be achieved with plasmapheresis alone or in combination with other immunotherapies [60].

## 14. Plasmapheresis in Opsoclonus-Myoclonus

This group represents a collection of disorders in young children sharing autoimmune neurological manifestations with or without associated neoplasm. The common presentation often shows in form of imbalance, ataxia, drooling, mood changes, tremors, rotator eye movements, and myoclonus [61]. The most common concurrent neoplasm is neuroblastoma [61]. Presence of autoantibodies have been documented, but not in all patients [62]. Luckily children tend to improve significantly after removal or treatment of neuroblastoma, unlike adults [63]. The response to plasmapheresis is among the best clinical responses, as compared to other autoimmune neurological disorders discussed above [64, 65]. Moreover, plasmapheresis can also help in alleviating relapse symptoms in recurrent opsoclonus-myoclonus syndrome [66].

## 15. Plasmapheresis in Nonencephalopathic Neurological Disorders

Usefulness of plasmapheresis is not limited to AIE diseases. There are other potential utilities in variety of neuromuscular autoimmune disorders. Some examples are Guillain Barre syndrome (GBS), myasthenic syndromes, peripheral neuropathies, and others. Obviously, many nonneurological indications are present as well. As dissecting such disease categories are outside the scope of this paper about AIE, they will not be discussed in detail.

## 16. Potential Challenges in Plasmapheresis

Plasmapheresis is not a simple treating procedure. There are a lot of difficulties across logistic, clinical, and patients aspects. Being a unique service, availability is always a major challenge even in well developed healthcare systems. The human resources are another big dilemma. Specialized trained physicians and technicians with extensive relevant experience are usually on shortage. Plasmapheresis has to be delivered through a large venous access device, usually central lines. Suitability for such procedure is usually an issue and most centers have variable different cut of age, below which patients cannot be treated by plasmapheresis. Even in older children, sedation and cooperation are seldom an easy task, making compliance rates lower than actual patients' recruitment numbers. Successful run of plasmapheresis sessions is not without complications. Serious electrolyte disturbances are not uncommon but usually are manageable with appropriate replacement therapy [67]. Hypothermia is a frequent complication as well and warmers should be kept ready for usage [67]. As for other blood products transfusion, risk of allergic reaction of all grades should be kept on mind so urgent medical management can be delivered should any reaction happens. With large volume pheresis manifestations of hypocirculation might be encountered. Coagulation profile alteration is a known complication; thus regular coagulation markers monitoring is a must. Risk of procedure-borne infection is present. However, with current rather standard blood banks procedure the risk is relatively small.

## 17. Conclusion

Plasmapheresis is an essential and perhaps competing as a potential first-line immunotherapy for various subtypes of autoımmune encephalopathy (AIE). Entertaining autoimmune medicated encephalopathy as a potential diagnosis is vital to direct early and efficient utilization of plasmapheresis in such settings. Accumulating experience and evidence behind plasmapheresis might shift the procedure to be a cornerstone first-line treatment option for children with autoimmune encephalopathy in the near future. We have reviewed some of the possible clinical applications for plasmapheresis in children with autoimmune encephalopathy. We believe this review cannot serve as expanded reference for the available evidence behind plasmapheresis in pediatric autoimmune encephalopathy. However, it can work as a quick summarized review of the most recent and up-to-date relevant scientific knowledge. Obviously, more clinical trials are required for strengthening our evidence class, enriching our current knowledge, and improving our procedural practice of this possibly lifesaving treatment modality.
